# Comparative mitogenomics analysis of *Peltigera* species and new insights into the lichen phylogenetics

**DOI:** 10.3389/fmicb.2025.1599036

**Published:** 2025-07-15

**Authors:** Reyim Mamut, Gulbar Yisilam, Guldiyar Adil, Gulmira Anwar

**Affiliations:** College of Life Sciences and Technology, Xinjiang University, Urumchi, China

**Keywords:** *Peltigera*, mitochondrial genome, repeat sequences, genomics, phylogeny

## Abstract

The genus *Peltigera* includes terricolous and muscicolous foliose macrolichens that are common and widespread across the majority of continents. The genus is well-defined by the absence of a lower cortex and the presence of a dense arachnoid-tomentose pilema that usually bears anastomosing pale or dark veins with numerous solitary or confluent rhizines. However, the high morphological similarity among its species poses significant taxonomic challenges for accurate identification, often necessitating the use of molecular data. This study presents a comprehensive mitogenomic analysis of 11 *Peltigera* species, with the aim of elucidating their genetic structure and taxonomic status. We sequenced and performed *de novo* assembly of the complete mitochondrial genomes, followed by comprehensive genomic analysis. This analysis revealed that the circular genome length ranged in length from 52,107 bp to 76,353 bp with guanine and cytosine (GC) contents between 26.4 and 27.4%. The mitogenomes encoded 15 protein-coding genes (PCGs), 26–27 transfer RNAs (tRNAs), and 2 ribosomal RNAs (rRNAs). Our analysis revealed a variety of scattered repeats, simple repeats, and tandem repeats, predominantly in intergenic and intron regions. Additionally, we examined codon usage and conducted a synteny analysis within the mitogenomes of *Peltigera* species. These findings enhance our understanding of the genetic evolution and phylogenetic relationships within *Peltigera*, providing a scientific foundation for future research on the diversity and evolution of lichenized fungi.

## Introduction

Phylogenomic datasets continue to enhance our understanding of evolutionary relationships in various lineages of organisms. However, genome-scale data have not been widely used to reconstruct relationships in lichenized fungi (Pizarro et al., 2018). Lichens serve as ideal model systems for testing these hypotheses because several genera and species are globally distributed and form locally differentiated subgroups (Fernández-Mendoza et al., 2011). Lichens have evolved independently over 10 times in the fungal tree of life (Jones et al., 2015), exemplifying convergent evolution as they perform similar functions across unrelated lineages (Allen and Lendemer, 2022). The main symbiotic relationship is formed by a fungus (mycobiont) and green algae and/or cyanobacteria (photobiont). Furthermore, the lichen thallus includes a diverse community of associated bacteria, algae, endolichenic or lichenicolous fungi, and basidiomycete yeasts (Ruprecht et al., 2020). The systematic study of lichens has followed a long yet continuous and stable progression. Beginning with the discovery of the dual nature of lichens, followed by the application of chemical components in taxonomy, and more recently, the clarification of lichen symbiosis and its constituent elements, these advancements have made significant contributions to the fields of symbiotic biology, physiology, and ecology. The rapidly advancing field of lichenology, like research on other biological groups, faces numerous challenges. From a taxonomic perspective, groups characterized by complex chemistry, highly variable or similar external morphology, and a lack of stable morphological diagnostic features present particular challenges. Consequently, methodological innovations in lichen taxonomy are urgently needed. With the continuous advancement of cutting-edge technologies, including restriction enzymes, PCR, DNA sequencing, and high-throughput sequencing, lichenologists have recognized that analyzing genetic variation in individual genes is insufficient. Significant uncertainties remain when inferring geographic origins and reconstructing phylogenetic relationships based on single sequence markers. Current research demonstrates that mitochondrial genomes show exceptional utility in resolving higher taxonomic relationships (e.g., ordinal-level differentiation in mammals) and species complexes (e.g., in insects and fishes). These findings indicate that mitochondrial phylogenomics is evolving from a supplementary tool into a cornerstone of evolutionary research. Applying mitochondrial genomics to lichenological studies will not only address taxonomic challenges in complex groups but also advance research in lichen evolution, ecology, and functional genomics, bearing both significant theoretical and applied value.

*Peltigera* Willd., 1787, a genus of lichen-forming fungi (mycobionts), associates with cyanobacteria (cyanobionts) primarily from the genus *Nostoc* (Magain et al., 2018). This genus comprises terricolous and muscicolous foliose macrolichens that are common and widely distributed across most continents, and it exhibits extensive morphological and chemical variation both within and between species, posing significant challenges for species delimitation and identification (Magain et al., 2010; Miadlikowska et al., 2014). *Peltigera* is a typical foliose lichen; it is easily identifiable in the field by the absence of a lower cortex and the presence of a dense arachnoid-tomentose pilema that usually bears anastomosing pale or dark veins with numerous solitary or confluent rhizines. However, Interspecific delimitation within *Peltigera* is difficult due to high phenotypic convergence, as traditional morphological and chemical characters often fail to resolve taxonomic ambiguities (Magain et al., 2018; Miadlikowska et al., 2018). For example, similar thallus structures and secondary metabolite profiles among species make it difficult to distinguish closely related taxa based on morphology alone (Magain et al., 2018). This issue is further complicated by the fact that morphological traits can be highly plastic and influenced by environmental conditions (Smith and Hill, 2019). As a result, molecular techniques, particularly multi-gene phylogenetic analyses, have been widely adopted as standard methodologies in research studies of this genus over the past decade (Miadlikowska et al., 2014, 2018; Wijayawardene et al., 2022). However, these studies have often been limited by insufficient taxon sampling and the use of a relatively small number of molecular markers, leading to persistent phylogenetic uncertainties. Recent studies have revealed an unexpectedly high species richness in *Peltigera*, with 34 species identified in the dolichorhizoid and scabrosoid clades of Polydactylon section, including 24 species new to science (Magain et al., 2023). Such findings highlight the complexity and underexplored nature of this genus, underscoring the need for more comprehensive phylogenetic analyses.

In fungi, mitogenomes exhibit intermediate mutation rates between those of plants and animals, making them particularly useful for resolving mid-to-deep-level phylogenetic relationships (Zhang et al., 2015). Prior studies have reported mitogenome sizes ranging from 52 to 76 kbp in *Peltigera*, with significant variation attributed to intron proliferation and repetitive element expansion (Xavier et al., 2012; Anwar et al., 2023). Beyond structural variation, codon usage bias in mitochondrial genes may reflect evolutionary adaptations to symbiotic niches. In free-living fungi, codon preferences are often shaped by mutational bias and translational selection (Arella et al., 2021). However, in lichenized fungi like *Peltigera*, the interplay between host and symbiont genomes may impose additional constraints on mitochondrial gene expression, potentially favoring AT-rich codons to optimize translational efficiency under symbiotic conditions (Beaudet et al., 2013). Comparative studies have shown that fungal mitogenomes exhibit intermediate mutation rates between those of plants and animals (Fonseca et al., 2021), but the specific phylogenetic relationships and structural characteristics of *Peltigera* mitogenomes remain unclear. This knowledge gap limits our ability to understand the evolutionary history and diversification within this genus fully.

This study addresses these knowledge gaps through comparative mitogenomic analysis of 16 *Peltigera* species (11 newly sequenced and 5 previously published), mainly focused on (1) analyzing the mitogenome structural characteristics of *Peltigera*; (2) evaluating the congruence between mitochondrial and nuclear phylogenies to test Miadlikowska’s classification (2000); and (3) contributing to a higher level classification for all groups of lichenized fungi in light of recent molecular phylogenetic studies.

## Materials and methods

### Sample collection and identification

The selection strategy of this study integrates taxonomic coverage (representing different evolutionary clades), ecological representativeness (adapting to diverse habitats in Xinjiang), and geographic uniqueness (Central Asian Transitional Zone). It aims to reveal the evolutionary dynamics of the genus *Peltigera* through comparative mitochondrial genomics and to provide new data for the study of the symbiotic mechanisms of lichens globally. The 11 *Peltigera* species were collected from Xinjiang, Northwest China. The species information is provided in Supplementary Table S1. The specimens were deposited in the Herbarium of the College of Life Science and Technology of Xinjiang University (XJU). The species were identified through a combination of their morphology, internal anatomy, and chemical characteristics. (1) The shape and color of the thallus: the smoothness of the upper surface was examined under a dissecting microscope; the unique cephalodia of the lichens (soredia, isidia, tomentum); the shape, thickness and color of the veins; the shape and color of the rhizine, and the size, color, and shape of the apothecium were observed using a dissecting microscope. (2) The number, shape, size, and type of photobiont (green algae or cyanobacteria) of the apothecium were observed using a stereoscopic microscope. (3) Color-developing reagents were applied to the thallus cortex. The color change was observed immediately, and the results were compared with standard color reaction charts for lichen chemical tests.

### DNA extraction and sequencing results

Approximately 1 cm of apothecium or thallus was rinsed three times with distilled water to remove impurities and then dried in a 1.5 mL centrifugal tube. The sample was homogenized into a fine powder using a high-throughput tissue grinder. DNA was extracted using the EZUP column fungal genomic DNA extraction kit (Sangon Biotech, Shanghai, China), following the manufacturer’s instructions. After extraction, the quality of the genomic DNA was assessed, and the DNA was stored at −20°C refrigerator. Whole-genome sequencing (WGS) of the *Peltigera* species was conducted using the DNBSEQ sequencing platform (Shenzhen, China).

### Mitochondrial genome assembly and annotation

The mitogenome assembly was performed using GetOrganelle V1.7.4.1 (Jin et al., 2020) and NOVOPlasty V4.2 (Dierckxsens et al., 2017). Subsequently, the assembled sequences were annotated using online tools GeSeq V2.03 (Tillich et al., 2017), MFannot V1.3.3 (Valach et al., 2014) and Mitos 2 (Bernt et al., 2013) (https://usegalaxy.eu). Next, the annotation results were verified in Geneious V2022.1.1 (Kearse et al., 2012) and the initiation and termination codons of the protein-coding gene (PCGs) were manually adjusted. Finally, the mitogenome map was generated using OGDraw V1.2 (Lohse et al., 2007).

### Repetitive sequence analysis

This study analyzed different types of repetitive sequences in the mitogenomes of 11 species, including interspersed repeats, tandem repeats, and simple sequence repeats (SSRs), to evaluate their types and distribution. Basic Local Alignment Search Tool (BLASTn) searches (Chen et al., 2015) were performed for each mitogenome against itself. Online software Tandem Repeats Finder V4.0 (Benson, 1999) (https://tandem.bu.edu/trf/trf.html) was employed to detect tandem repeats. REPuter (Kurtz et al., 2001) (https://bibiserv.cebitec.uni-bielefeld.de/reputer) was used to detect interspersed repeats, with the Hamming distance set to 3, the maximum computed repeats set to 5,000, and the minimal repeat size set to 30. MIcroSAtellite Identification Tool (MISA; Beier et al., 2017) (https://webblast.ipk-gatersleben.de/misa/) was used to detect simple sequence repetitions (SSRs). The motif size of one-to-six-nucleotide SSRs was set to 10, 5, 4, 3, and 3, respectively. Finally, TBtools (Chen et al., 2020) was used to visualize the distribution results of repetitive sequences.

### Codon usage analysis

For codon usage analysis, the conserved PCGs (*atp6*, *atp8*, *atp9*, *cytb*, *cox1*, *cox2*, *cox3*, *nad1*, *nad2*, *nad3*, *nad4*, *nad4L*, *nad5*, and *nad6*) of 11 *Peltigera* species were extracted using PhyloSuite V1.2.2 (Zhang et al., 2020). Subsequently, the relative synonymous codon usage (RSCU) was calculated using MEGA V11 (Tamura et al., 2021).

### Comparative mitochondrial genome analysis

The comparative genomics analysis was conducted on 16 mitogenome sequences, comprising 11 newly assembled sequences and five previously published sequences (Supplementary Table S2) (Anwar et al., 2023). The selection of these 16 species was based on their taxonomic representation and availability in public databases. The previously sequenced genomes were used as references to provide a broader context for the newly sequenced mitogenomes and to enhance the robustness of the comparative analysis. Mauve V2.4.0 (Darling et al., 2004) was used for collinearity analysis among the 16 species.

### Phylogenetic analysis

A total of 21 mitogenomes were used to determine the phylogenetic position of *Peltigera* species, with *Pertusaria propinqua* selected as the outgroup. The 21 species included the 16 species from the comparative analysis and five additional species chosen based on their taxonomic significance and availability in public databases. The previously sequenced genomes were used as references to provide a comprehensive phylogenetic framework. The 14 mitochondrial PCGs, including *atp6*, *atp8*, *atp9*, *cytb*, *cox1*, *cox2*, *cox3*, *nad1*, *nad2*, *nad3*, *nad4*, *nad4L*, *nad5*, and *nad6*, which are conserved across the 21 analyzed species, were aligned using MAFFT V7.313 (Katoh et al., 2019) with default parameters, followed by missing data elimination using Gblocks V0.91b (Castresana, 2000) with default parameters. Subsequently, a maximum likelihood (ML) tree was constructed in IQ-tree V1.6.8 (Nguyen et al., 2015) with the parameters -m MFP -bb 1,000 -nt AUTO. Finally, FigTree V1.4.3 was used to visualize the phylogenetic tree.

## Results

### Features of the newly assembled *Peltigera* mitogenome

**Figure 1:**
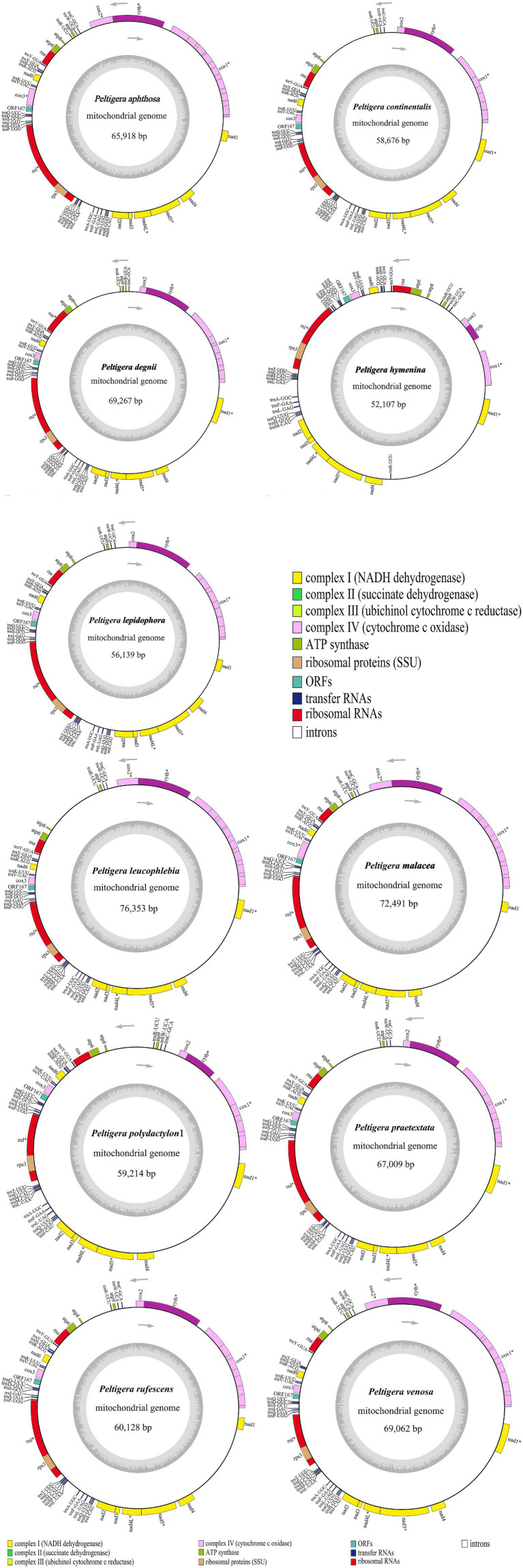
Circular maps of 11 *Peltigera* mitogenomes. Genes with different functions are represented by different colors. The genes inside the circle are on the direct strand, and the genes outside the circle are on the reverse strand. *Genes with introns.

The complete mitogenomes of 11 *Peltigera* species, as depicted in Figure 1, varied in size from 52,107 to 76,353 base pairs, with GC contents ranging from 26.4 to 27.4%. These mitogenomes contained 14 PCGs: *atp6*, *atp8*, *atp9*, *cytb*, *cox1*, *cox2*, *cox3*, *nad1*, *nad2*, *nad3*, *nad4*, *nad4L*, *nad5*, and *nad6*, along with 26–27 transfer RNAs (tRNAs) and 2 ribosomal RNAs (rRNAs). Detailed proportions of coding and non-coding regions are provided in Supplementary Table S3. Among these 14 PCGs, the *cox1* gene contained the highest number of introns, with copy numbers ranging from 1 to 9. The 11 newly generated sequences from this study have been submitted to the GenBank database with accession numbers PP740889 to PP740899.

The longest intergenic regions between the *nad4* and *nad1* genes were found in *Peltigera aphthosa*, *Peltigera degenii*, *Peltigera hymenina*, *Peltigera malacea*, *Peltigera leucophlebia*, *Peltigera polydactylon*, *Peltigera praetextata*, *Peltigera rufescens*, and *Peltigera venosa*. The longest intergenic regions between the *trnR* and *atp8* genes were found in *Peltigera continentalis* and *Peltigera lepidophora*.

### Repetitive element analysis

**Figure 2:**
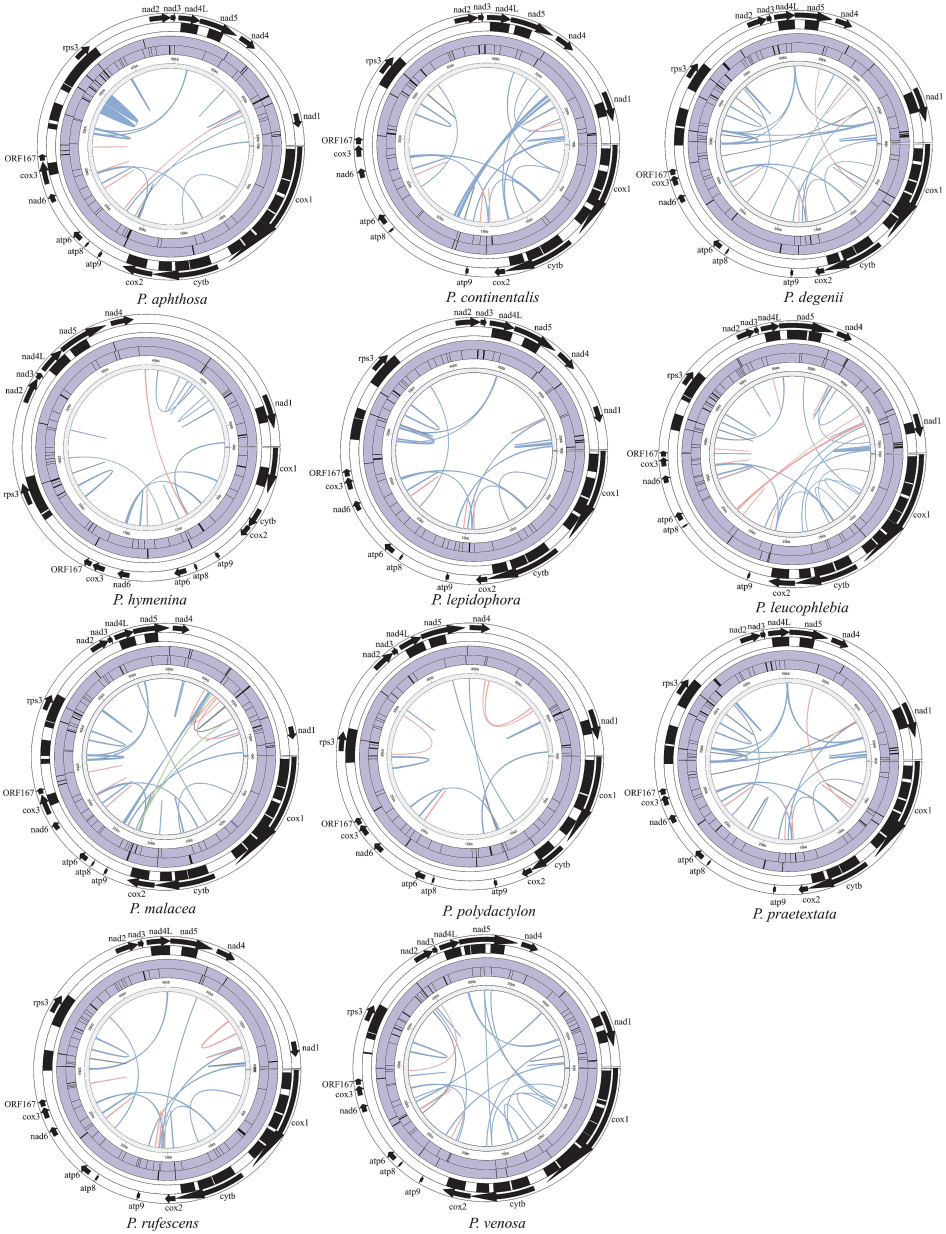
The repeated distribution map of 11 *Peltigera* mitogenomes. Each circle from inside to outside represents the following: Interspersed repeats (blue represents “Forward,” pink represents “Palindromic,” gray represents “Reverse,” and green represents “Complement”), SSRs, tandem repeats, introns, mitogenomes coding region. The black square indicates the area covered by each element, while the purple filling is not relevant.

We identified various types of repetitive sequences, including interspersed repeats, simple sequence repeats (SSRs), and tandem repeats, in the mitogenomes of 11 species of *Peltigera*. In this study, the number of interspersed repeats varied from 27 to 270 across the 11 species, with sizes ranging from 30 to 1,501 bp (Supplementary Tables S4–S14). These interspersed repeats consisted of 20–86 forward repeats, 3–66 palindrome repeats, and 1–75 reverse repeats. Additionally, 33–55 simple sequence repeats (SSRs) were identified, ranging in size from 10 to 217 bp. These SSRs included 19–30 mononucleotides, 5 bp to 14 dinucleotides, 2–10 trinucleotides, 2–7 tetranucleotides, 1–3 pentanucleotides, and 1–4 hexanucleotides. Furthermore, 8–26 tandem repeats were identified, with repeat unit sizes ranging from 2 to 124 bp and copy numbers varying from 1 to 35, as illustrated in Figure 2.

### Codon usage analysis

**Figure 3:**
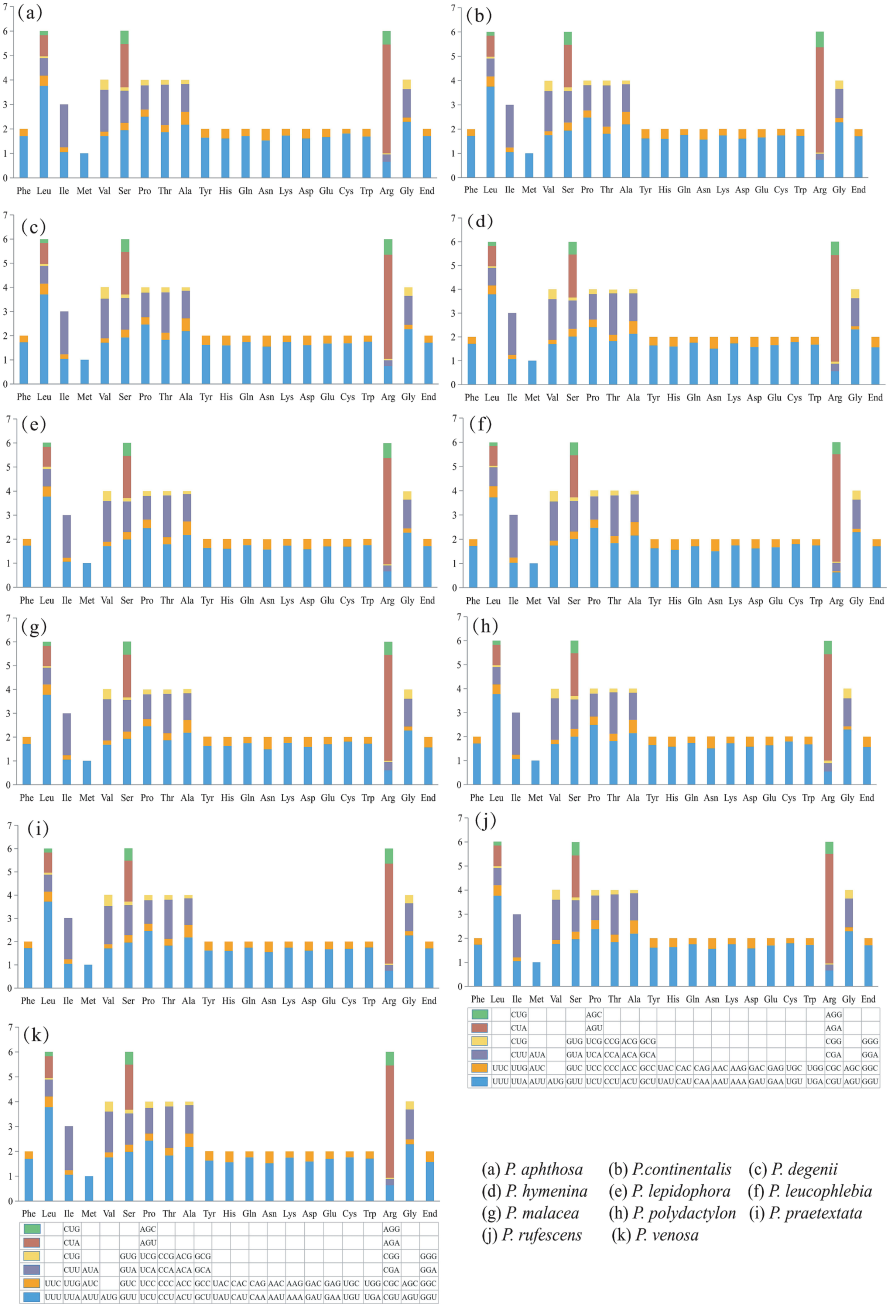
Codon usage analysis of 11 *Peltigera* species mitogenomes. The *x*-axis comprises the 20 standard amino acids that encode the protein, and the encoding codon is featured below each amino acid. The *y*-axis is the frequency of codon usage.

The results of the codon usage analysis of the PCGs showed that among the 64 codons encoding 20 amino acids, leucine (Leu), isoleucine (Ile), Serine (Ser), and Phenylalanine (Phe) were the most frequently used amino acids. The codons with the highest relative synonymous codon usage (RSCU) were AGA, UUA, CCU, GGU, and GCU. The codon preferences of the PCGs in the mitogenomes of the 11 species were highly similar. Leucine (Leu), serine (Ser), and arginine (Arg) were encoded by six codons, while methionine (Met) was encoded by only one codon in the 11 *Peltigera* species mitogenome (Figure 3).

Most PCGs (*atp6*, *atp8*, *atp9*, *nad1*, *nad2*, *nad3*, *nad4*, *nad4L*, *nad5*, and *rps3* genes) in the mitogenomes of 11 species (Supplementary Table S15) had the typical ATG start codon. The initiation codon for the *cytb* gene was GTG in all species. The *cox1* gene used ATG, and only the *cox1* gene of *P. praetextata* used ATA as the starting codon. The *cox2* gene used ATT as the initiation codon. The *nad6* genes of most species used ATA as the initiation codon, and a few *nad6* genes used ATG as the initiation codon. Analysis of codon preference in the 11 species showed that each species had 64 codons, 7 species (*P. continentalis, P. degenii, P. hymenina, P. malacea, P. praetextata, P. rufescens*, and *P. venosa*) 28 codons had RSCU values There are 35 codons with RSCU values <1, 16 of which end in C and 15 in G. Four species (*P. aphthosa*, *P. lepidophora*, *P. leucophlebia*, and *P. polydactylon*) had 27 codons with RSCU values >1, 13 of which end in A and 14 of which end in U. In total, 36 codons had RSCU values <1; 16 of them ended in C and 15 in G. Methionine (Met) had an RSCU value of 1, and there was no codon usage preference.

### Comparative analysis

We compared genome sizes, GC contents, PCGs, rRNAs, and tRNAs, as well as the lengths of coding, intergenic, intronic, and RNA regions in each mitogenomes of 16 *Peltigera* species.

**Figure 4:**
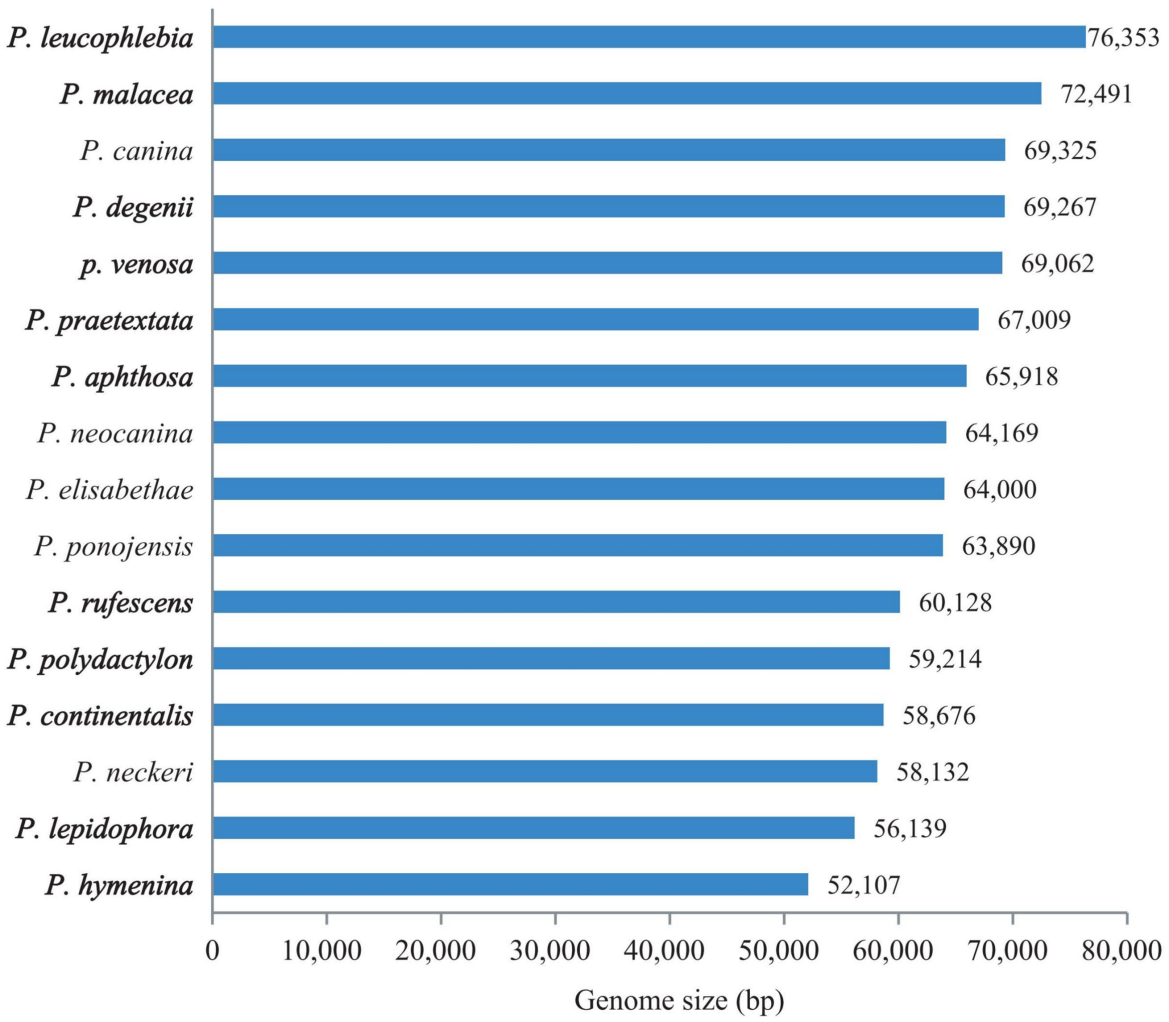
Comparison of the mitogenome length of 16 *Peltigera* species.

The mitogenome sizes of the 16 *Peltigera* species exhibited variation (Figure 4). *P. leucophlebia* possessed the largest mitogenomes, measuring 76,353 bp, followed by *P. malacea*, while the smallest was 52,107 bp, found in *P. hymenina*. The GC content among these species was relatively consistent, ranging from 26.4% in *P. degenii* and *P. neocanina* to 27.4% in *P. aphthosa*. The lengths of the PCGs and RNA genes were relatively uniform across the 16 mitogenomes; however, the lengths of the non-coding regions, including intergenic and intronic regions, showed significant variation among species. Specifically, the PCGs ranged in length from 13,631 to 14,169 bp, the RNA genes varied from 5,782 to 6,967 bp, the intron regions spanned from 8,207 to 26,929 bp, and the intergenic regions extended from 17,025 to 28,856 bp.

**Figure 5:**
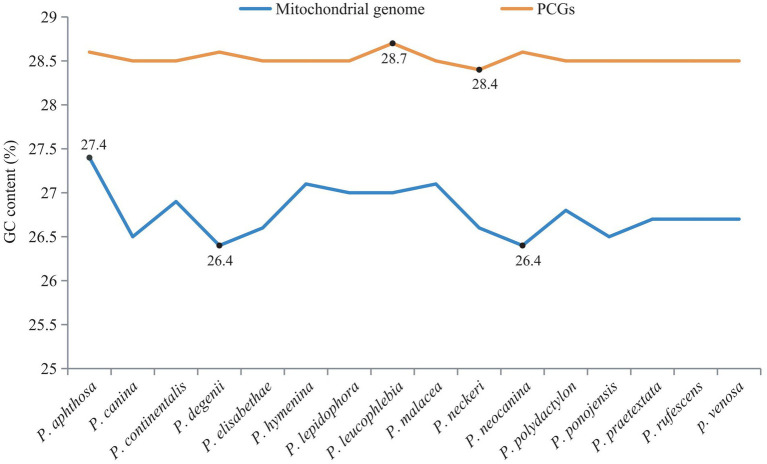
Comparison of GC content in the mitogenome of 16 *Peltigera* species.

The GC contents of the mitogenome sequences and 15 PCG sequences were calculated (Figure 5). There was little difference in the GC content between the whole mitogenomes and PCGs. The GC contents of the PCGs ranged from 28.4% (*Peltigera neckeri*) to 28.7% (*P. leucophlebia*). The GC contents of the whole mitogenomes ranged from 26.4% (*P. degenii* and *P. neocanina*) to 27.4% (*P. aphthosa*). The mitogenomes and PCGs of the 16 *Peltigera* species exhibited a high adenine and thymine nucleotide (AT) content, which was much higher than the GC content.

**Figure 6:**
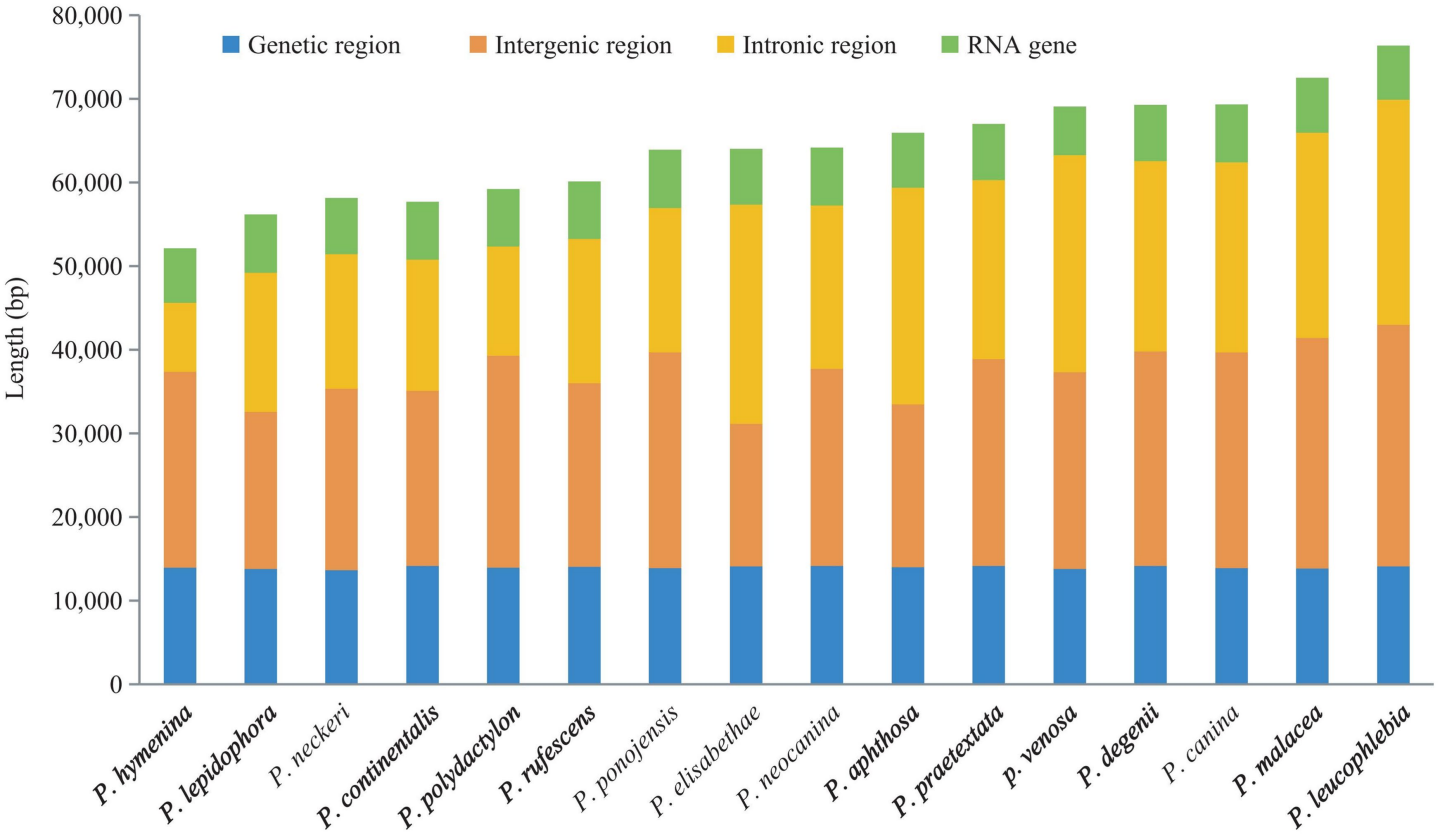
Proportions of the protein-coding, intergenic, intronic, and RNA regions in the 16 *Peltigera* mitogenomes.

To investigate the conservation of coding and non-coding region sizes in the mitogenomes of *Peltigera* and their relationship to the overall mitogenome size (as shown in Figure 6), we quantified the lengths of the coding, intergenic, intronic, and RNA (tRNA and rRNA) regions in each mitogenome. The sizes of the PCGs and RNA genes were found to be relatively conserved across the mitogenomes, whereas the non-coding regions, which include intergenic and intronic regions, exhibited substantial variation both within and between species. Specifically, the lengths of the protein-coding regions in the 16 mitogenomes ranged from 13,631 to 14,169 bp. The RNA regions spanned 5,782 to 6,967 bp. The intron regions varied from 8,207 to 26,929 bp, and the intergenic regions ranged from 17,025 to 28,856 bp.

**Figure 7:**
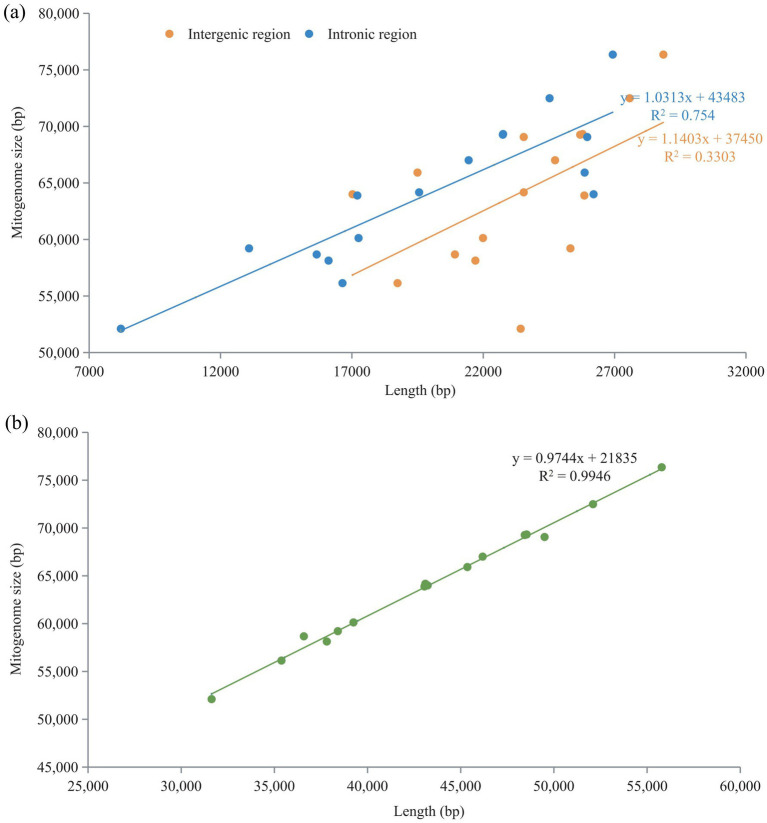
Pearson correlation analysis of intronic and intergenic regions with genome size. **(a)** Relationship between intergenic and intronic regions and mitogenome size. **(b)** Relationship between intronic and intergenic regions and mitogenome size.

Pearson correlation analysis revealed a strong positive correlation between the sizes of the intronic and intergenic regions and the overall size of the mitogenome, with R^2^-values of 0.754 and 0.3303, respectively (Figure 7a). The correlation coefficient for the relationship between intron length and mitogenome size was particularly high, at R^2^ = 0.9946. This indicates that the longer the introns, the larger the mitogenomes tend to be. Conversely, a weaker positive correlation was observed between the size of the intergenic region and the mitogenome size, with an R^2^-value of 0.2401 (Figure 7b). This suggests that while a relationship exists, a longer intergenic region does not predict a larger mitogenome size as strongly as the length of introns.

### Synteny analysis

**Figure 8:**
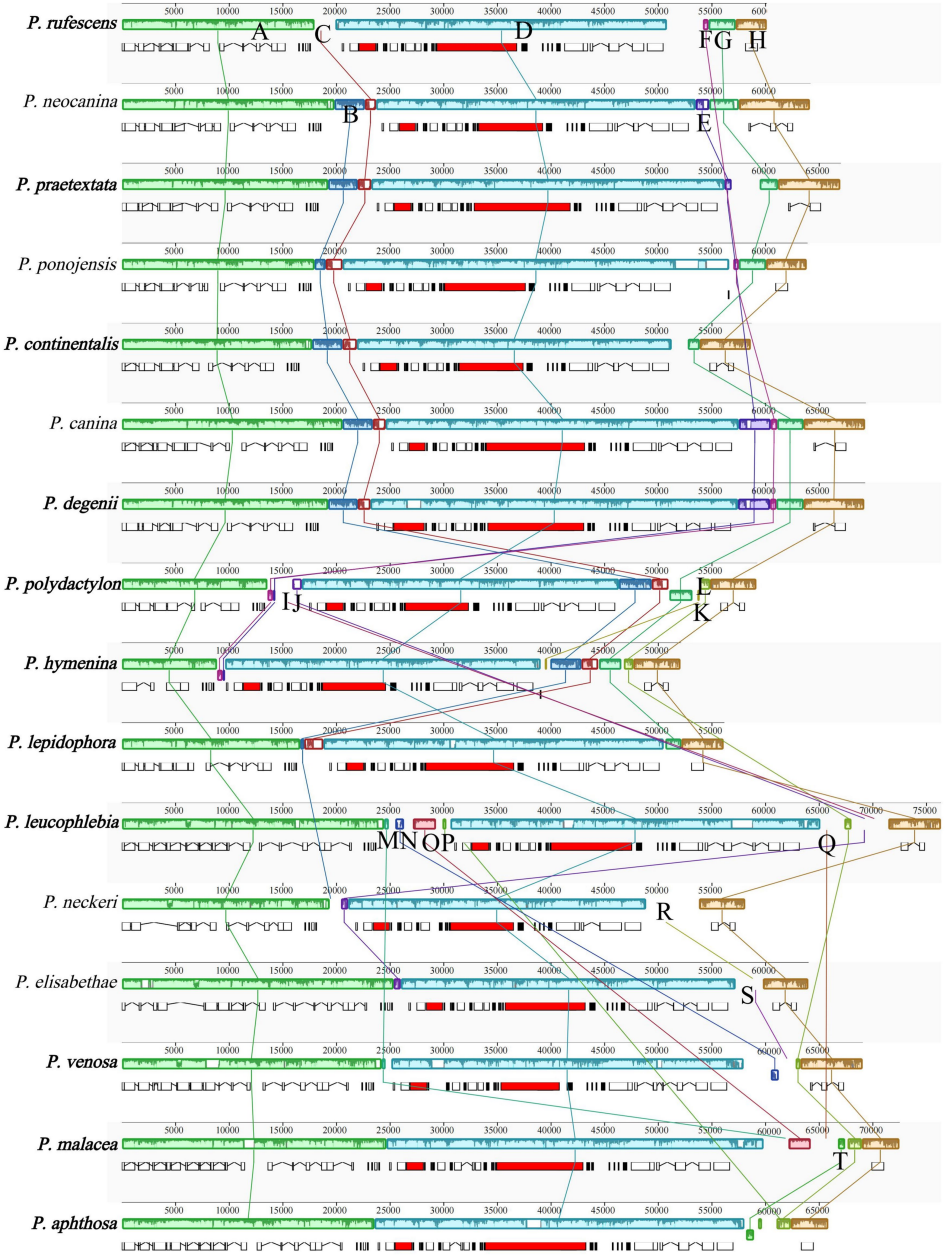
Comparative mitogenomic gene rearrangement analysis of the 16 *Peltigera* species using Mauve. The same color blocks represented homologous regions between different species. Species in this study are shown in bold.

A total of 20 locally collinear blocks (A-T) were identified in the mitogenomes of the 16 *Peltigera* species (as shown in Figure 8). Among these, collinearity blocks A, D, and H were present in all 16 mitogenomes. Collinearity block B was found in 10 mitogenomes, with the exception of *P. rufescens*, *P. leucophlebia*, *Pseudopterogorgia elisabethae*, *P. venosa*, *P. malacea*, and *P. aphthosa*. Collinearity block C was present in 11 mitogenomes, excluding *P. leucophlebia*, *P. neckeri*, *P. elisabethae*, *P. venosa*, *P. malacea*, and *P. aphthosa*. Collinearity block E was observed in *P. neocanina*, *P. praetextata*, *P. canina*, *P. degenii*, *P. polydactylon*, and *P. hymenina*. Collinearity block F was detected in *P. rufescens*, *P. ponojensis*, *P. canina*, *P. degenii*, *P. polydactylon*, and *P. hymenina*. Collinearity block G was found in the mitogenomes of species from Sections E and A. Collinearity block I was exclusively present in *P. polydactylon* and *P. leucophlebia*. Collinearity block K was only found in species from Section A. Collinearity block L was present in species from Sections A, B, C, and G. Collinearity block M was observed in species from Sections B and G, as well as in *P. malacea*. Collinearity block N was exclusively found in species from Sections B and G. Collinearity block O was only present in the mitogenomes of *P. leucophlebia* and *P. malacea*. Collinearity block P was only found in *P. leucophlebia* and *P. aphthosa*. Collinearity block Q was only present in *P. leucophlebia* and *P. malacea*. Collinearity block T was exclusively found in *P. aphthosa* and *P. malacea.*

### Phylogenetic analysis

**Figure 9:**
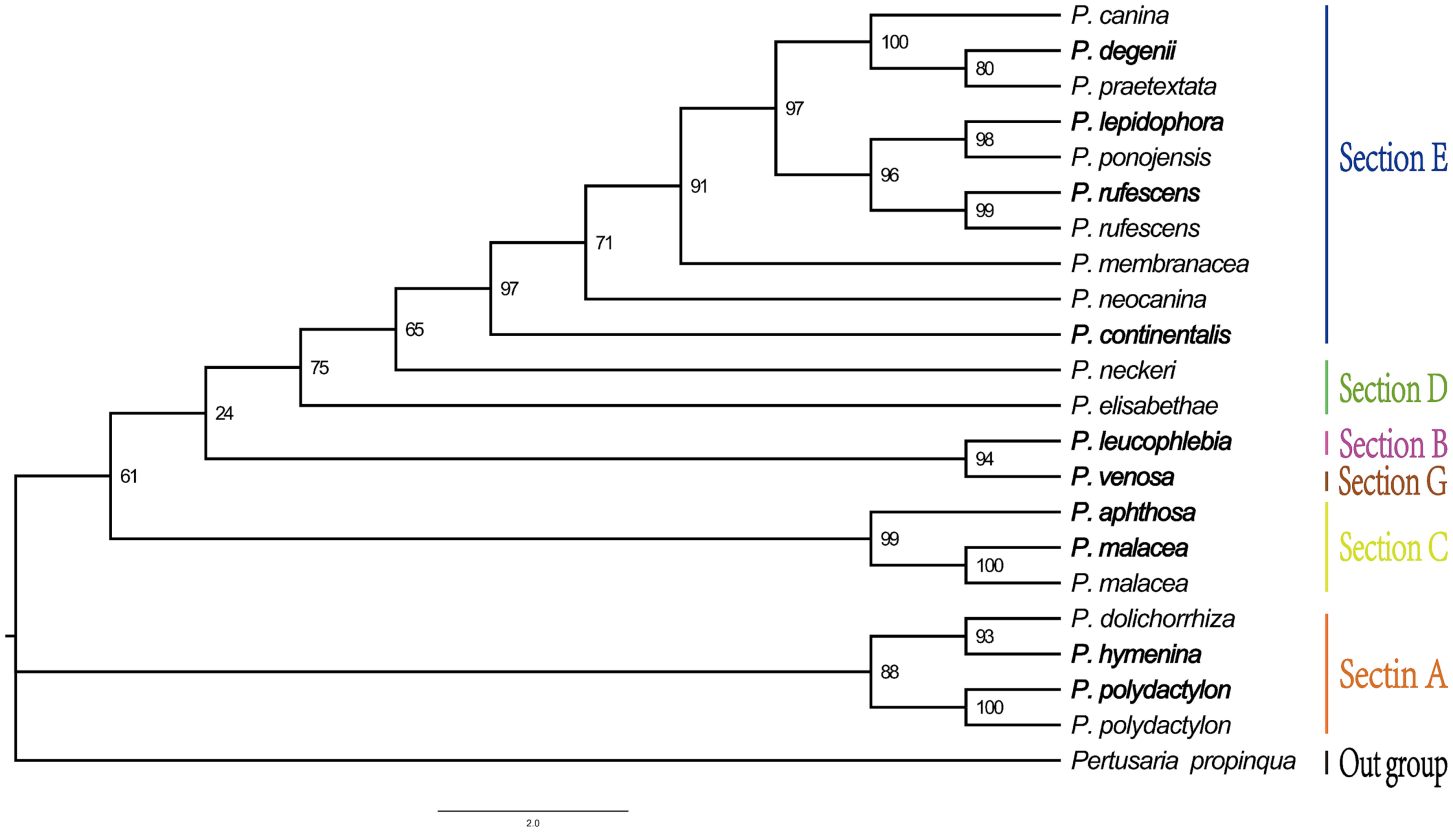
The phylogenetic tree of 21 *Peltigera* species based on the 14 PCGs.

The maximum likelihood (ML) tree, constructed based on mitogenome sequences, divided the 21 *Peltigera* species into six sections with high bootstrap support values (Figure 9). The phylogenetic analysis results indicated that the species were divided into two major branches. The first branch comprised sections E and D, while the second branch included sections A, G, B, and C. *P. hymenina* and *Peltigera dolichorhiza*, both in section A, formed a sister branch with the Polydactylon section and were clustered together with *P. venosa* from section G.

## Discussion

This study presents the first comprehensive analysis of mitogenomes from 11 *Peltigera* species in Xinjiang, Northwest China. Our assembled mitogenomes share conserved features with previous studies (Xavier et al., 2012), including 14 PCGs, 2 rRNAs, 26–27 tRNAs, and a circular structure. However, we identified a notable discrepancy in the mitogenome size of *P. malacea* (72.4 kbp vs. 63.4 kbp reported by Xavier et al., 2012). This variation may arise from methodological differences (e.g., improved next-generation sequencing (NGS) assembly accuracy vs. Sanger sequencing; Gut, 2013) or biological factors such as intraspecific divergence or cryptic speciation (Matute and Sepulveda, 2019). Further sampling across geographic ranges could clarify whether this size difference reflects technical limitations or true genomic diversity.

The identification of introns within specific genes, including *cox*1, *cytb*, *cox*2, *nad*4L, *nad*5, and *nad*1, as well as the *rnl* and *rns* rRNA genes, among the 14 PCGs, adds an intriguing layer of complexity to our understanding. Introns, intergenic regions, and repetitive sequences significantly impact the fungal mitogenome size (Fonseca et al., 2021; Zhang et al., 2015). In *Peltigera*, intron lengths range from 8,207 to 26,929 bp (15.75–40.94% of the mitogenome), intergenic region lengths from 17,025 to 28,856 bp (26.60–44.95% of the mitogenome), and repetitive sequences display diverse features (27–270 interspersed repeats of 30–1,501 bp with specific sub-types, 33–55 SSRs of 10–217 bp with various nucleotide compositions, and 8–26 tandem repeats of 2–124 bp with 1–35 copy numbers). These data suggest that Peltigera is subject to unique evolutionary pressures or genomic architectures. Compared to free-living fungi with simpler structures, the longer gene lengths in *Peltigera* suggest more complex regulatory requirements. The variations within *Peltigera* influence gene regulation, recombination, and the evolution of the mitogenome. Comparisons with other symbiotic or non-symbiotic fungi, such as mycorrhizal fungi that interact with different hosts, reveal how *Peltigera* has adapted to its lichen symbiotic partners. For instance, mycorrhizal fungi often exhibit more compact mitogenomes with fewer introns and repetitive elements, which may reflect different selective pressures related to their symbiotic interactions with plant roots (Fonseca et al., 2021). In contrast, the larger and more complex mitogenomes of *Peltigera* could be an adaptation to the more intricate ecological niche of lichen symbiosis, where the fungus must coordinate with both a photobiont and other microbial partners. This complexity may also be related to the need for greater genetic flexibility to adapt to environmental changes (Smith and Hill, 2019).

Codon usage bias plays a critical role in protein translation, as it provides insights into species evolution and environmental adaptation (Arella et al., 2021; Beaudet et al., 2013). Within *Peltigera*, although variations exist in the utilization of initiation codons among different species, a remarkably consistent pattern of codon usage preference is evident. Specifically, codons such as AGA, UUA, CCU, GCU, and UGU are identified as the most frequently employed across the 11 mitogenomes. This pronounced preference for specific codons—particularly those enriched with adenine and uracil (A/U) bases—contributes to the elevated AT content observed in the *Peltigera* mitogenome. This characteristic may represent an adaptive response to their symbiotic lifestyle within lichens. It is plausible that this specific pattern of codon usage correlates with tRNA availability within these species. In the context of lichen symbiosis, this distinct codon usage could enhance translational efficiency under the unique conditions presented by this mutualistic relationship. When compared to non-symbiotic fungi, this adaptation in codon usage likely plays a pivotal role in facilitating both the establishment and maintenance of symbiotic states in *Peltigera* members. For example, a recent study on the mitogenome of the lichen-forming fungus *Cladonia rangiferina* also revealed a similar codon usage bias, suggesting that this may be a common feature among lichenized fungi (Jones et al., 2023).

In this study, the 21 species used to construct the phylogenetic tree, including the 16 available species from this study area, were divided into six sections: *Peltigera* sect. Polydactylon (section A), *Peltigera* sect. Chloropeltigera (section B), *Peltigera* sect. *Peltigera* (section C), and *Peltigera* sect. Horizontales (section D), *Peltigera* sect. *Peltigera* (section E), and *Peltigera* sect. Phlebia (section G) (Figure 9). Our analysis revealed that the phylogenetic tree topology received considerable support, with the majority of branches having bootstrap support values exceeding 70%, which is a threshold often considered to represent strong support in phylogenetic studies. The research results are consistent with those of the study by Miadlikowska et al. (2018). This study, therefore, provides a more comprehensive and robust phylogenetic framework for the genus *Peltigera*, highlighting the importance of mitogenomic data in resolving taxonomic uncertainties and clarifying evolutionary relationships within this complex genus. The inclusion of multiple species from different sections allows for a more detailed understanding of the phylogenetic structure and evolutionary dynamics within *Peltigera*.

Lichens, as ancient and classic symbiotic organisms, serve as excellent materials for investigating the origins of life on Earth and the dynamics of symbiotic relationships. Against the backdrop of rapid advancements in molecular biology and bioinformatics, research on lichens has become increasingly in-depth, and the field is currently in its initial stage of flourishing development. In terms of genomic research on lichen-forming fungi, although the data in databases have significantly increased in recent years, they remain minimal compared to the vast diversity of the lichen group. Integrated multi-omics research represents a significant trend in biological studies, and the use of multi-omics approaches will facilitate the exploration of lichen symbiotic mechanisms and evolutionary history. This disparity primarily stems from the complex structure of lichens and their relative neglect in research contexts. In extensively studied fungal models, such as *Neurospora crassa* or *Aspergillus nidulans*, comprehensive genomic datasets have facilitated detailed explorations into intron functionality and evolutionary dynamics. In comparison, our understanding of the mitogenomes of lichenized fungi remains in its early stages. Therefore, future research must prioritize addressing this data gap to elucidate the evolutionary characteristics of fungal mitogenomes associated with lichens. We believe that the mitogenomes of lichen-forming fungi will soon become a powerful tool for resolving challenges in phylogenetic studies.

## Data Availability

The datasets presented in this study can be found in online repositories. The names of the repository/repositories and accession number(s) can be found in the article/Supplementary material.

## References

[ref1] Allen J. L. Lendemer J. C. (2022). A call to reconceptualize lichen symbioses. Trends Ecol. Evol. 37, 582–589. doi: 10.1016/j.tree.2022.03.004, PMID: 35397954

[ref2] Anwar G. Mamut R. Wang J. Q. (2023). Characterization of complete mitochondrial genomes of the five *Peltigera* and comparative analysis with relative species. J. Fungi 9:969. doi: 10.3390/jof9100969, PMID: 37888225 PMC10607270

[ref3] Arella D. Dilucca M. Giansanti A. (2021). Codon usage bias and environmental adaptation in microbial organisms. Mol. Gen. Genomics. 296, 751–762. doi: 10.1007/s00438-021-01771-4, PMID: 33818631 PMC8144148

[ref4] Beaudet D. Terraty Y. Halary S. de la Providencia I. E. Hijri M. (2013). Mitochondrial genome rearrangements in *Glomus* species triggered by homologous recombination between distinct mtDNA haplotypes. Genome Biol. Evol. 5, 1628–1643. doi: 10.1093/gbe/evt120 23925788 PMC3787672

[ref5] Beier S. Thiel T. Munch T. Scholz U. Mascher M. (2017). MISA-web: a web server for microsatellite prediction. Bioinformatics 33, 2583–2585. doi: 10.1093/bioinformatics/btx198, PMID: 28398459 PMC5870701

[ref6] Benson G. (1999). Tandem repeats finder: a program to analyze DNA sequences. Nucleic Acids Res. 27, 573–580. doi: 10.1093/nar/27.2.573, PMID: 9862982 PMC148217

[ref7] Bernt M. Donath A. Jühling F. Externbrink F. Florentz C. Fritzsch G.. (2013). MITOS: improved metazoan mitochondrial genome annotation. Mol. Phylogenet. Evol. 69, 313–319. doi: 10.1016/j.ympev.2012.08.023 22982435

[ref9001] Castresana J. (2000). Selection of conserved blocks from multiple alignments for their use in phylogenetic analysis. Mol. Biol. Evol. 17, 540–552. doi: 10.1093/oxfordjournals.molbev.a026334 10742046

[ref8] Chen C. J. Chen H. Zhang Y. Thomas H. R. Frank M. H. He Y. H.. (2020). TBtools: an integrative toolkit developed for interactive analyses of big biological data. Mol. Plant 13, 1194–1202. doi: 10.1016/j.molp.2020.06.009, PMID: 32585190

[ref9] Chen Y. Ye W. C. Zhang Y. D. Xu Y. S. (2015). High speed BLASTN: an accelerated MegaBLAST search tool. Nucleic Acids Res. 43, 7762–7768. doi: 10.1093/nar/gkv784, PMID: 26250111 PMC4652774

[ref10] Darling A. C. E. Mau B. Blattner F. R. Perna N. T. (2004). Mauve: multiple alignment of conserved genomic sequence with rearrangements. Genome Res. 14, 1394–1403. doi: 10.1101/gr.2289704, PMID: 15231754 PMC442156

[ref11] Dierckxsens N. Mardulyn P. Smits G. (2017). NOVOPlasty: assembly of organelle genomes from whole genome data. Nucleic Acids Res. 45:e18. doi: 10.1093/nar/gkw955 28204566 PMC5389512

[ref12] Fernández-Mendoza F. Domaschke S. García M. A. Jordan P. Martínm M. P. Printzen C. (2011). Population structure of mycobionts and photobionts of the widespread lichen *Cetraria aculeata*. Mol. Ecol. 20, 1208–1232. doi: 10.1111/j.1365-294X.2010.04993.x 21324011

[ref13] Fonseca P. L. C. De-Paula R. B. Araujo D. S. Tome L. M. R. Mendes-Pereira T. Rodrigues W. F. C.. (2021). Global characterization of fungal mitogenomes: new insights on genomic diversity and dynamism of coding genes and accessory elements. Front. Microbiol. 12:787283. doi: 10.3389/fmicb.2021.787283, PMID: 34925295 PMC8672057

[ref14] Gut I. G. (2013). New sequencing technologies. Clin. Transl. Oncol. 15, 879–881. doi: 10.1007/s12094-013-1073-6, PMID: 23846243

[ref15] Jin J. J. Yu W. B. Yang J. B. Song Y. dePamphilis C. W. Yi T. S.. (2020). GetOrganelle: a fast and versatile toolkit for accurate de novo assembly of organelle genomes. Genome Biol. 21:241. doi: 10.1186/s13059-020-02154-5, PMID: 32912315 PMC7488116

[ref16] Jones E. B. G. Suetrong S. Sakayaroj J. Bahkali A. H. Abdel-Wahab M. A. Boekhout T.. (2015). Classification of marine Ascomycota, Basidiomycota, Blastocladiomycota and Chytridiomycota. Fungal Divers. 73, 1–72. doi: 10.1007/s13225-015-0339-4

[ref17] Jones A. A. Willoner T. Mishoe Hernandez L. DeLaurier A. (2023). Exposure to valproic acid (VPA) reproduces hdac1 loss of function phenotypes in zebrafish. MicroPubl. Biol. doi: 10.17912/micropub.biology.000908 PMC10565572 37829572

[ref18] Katoh K. Rozewicki J. Yamada K. D. (2019). MAFFT online service: multiple sequence alignment, interactive sequence choice and visualization. Brief. Bioinform. 20, 1160–1166. doi: 10.1093/bib/bbx108, PMID: 28968734 PMC6781576

[ref19] Kearse M. Moir R. Wilson A. Stones-Havas S. Cheung M. Sturrock S.. (2012). Geneious basic: an integrated and extendable desktop software platform for the organization and analysis of sequence data. Bioinformatics 28, 1647–1649. doi: 10.1093/bioinformatics/bts199, PMID: 22543367 PMC3371832

[ref20] Kurtz S. Choudhuri J. V. Ohlebusch E. Schleiermacher C. Stoye J. Giegerich R. (2001). REPuter: the manifold applications of repeat analysis on a genomic scale. Nucleic Acids Res. 29, 4633–4642. doi: 10.1093/nar/29.22.4633, PMID: 11713313 PMC92531

[ref21] Lohse M. Drechsel O. Bock R. (2007). OrganellarGenomeDRAW (OGDRAW): a tool for the easy generation of high-quality custom graphical maps of plastid and mitochondrial genomes. Curr. Genet. 52, 267–274. doi: 10.1007/s00294-007-0161-y, PMID: 17957369

[ref22] Magain N. Forrest L. L. Serusiaux E. Goffinet B. (2010). Microsatellite primers in the *Peltigera* dolichorhiza complex (lichenized ascomycete, Peltigerales). Am. J. Bot. 97, e102–e104. doi: 10.3732/ajb.1000283, PMID: 21616786

[ref23] Magain N. Miadlikowska J. Goffinet B. Goward T. Pardo-De la Hoz C. J. Jüriado I.. (2023). High species richness in the lichen genus *Peltigera* (*Ascomycota*, *Lecanoromycetes*): 34 species in the dolichorhizoid and scabrosoid clades of section *Polydactylon*, including 24 new to science. Persoonia 51, 1–88. doi: 10.3767/persoonia.2023.51.01, PMID: 38665978 PMC11041898

[ref24] Magain N. Truong C. Goward T. Niu D. L. Goffinet B. Sérusiaux E.. (2018). Species delimitation at a global scale reveals high species richness with complex biogeography and patterns of symbiont association in section (lichenized Ascomycota: Lecanoromycetes). Taxon 67, 836–870. doi: 10.12705/675.3

[ref25] Matute D. R. Sepulveda V. E. (2019). Fungal species boundaries in the genomics era. Fungal Genet. Biol. 131:103249. doi: 10.1016/j.fgb.2019.103249, PMID: 31279976 PMC7355355

[ref26] Miadlikowska J. Magain N. Pardo-De la Hoz C. J. Niu D. Goward T. Sérusiaux E.. (2018). Species in section *Peltidea* (*aphthosa* group) of the genus *Peltigera* remain cryptic after molecular phylogenetic revision. Plant Fungal Syst. 63, 45–64. doi: 10.2478/pfs-2018-0007

[ref27] Miadlikowska J. Richardson D. Magain N. Ball B. Anderson F. Cameron R.. (2014). Phylogenetic placement, species delimitation, and cyanobiont identity of endangered aquatic species (lichen-forming Ascomycota, Lecanoromycetes). Am. J. Bot. 101, 1141–1156. doi: 10.3732/ajb.1400267 25016011

[ref28] Nguyen L. T. Schmidt H. A. von Haeseler A. Minh B. Q. (2015). IQ-TREE: a fast and effective stochastic algorithm for estimating maximum-likelihood phylogenies. Mol. Biol. Evol. 32, 268–274. doi: 10.1093/molbev/msu300, PMID: 25371430 PMC4271533

[ref29] Pizarro D. Divakar P. K. Grewe F. Leavitt S. D. Huang J. P. Dal Grande F.. (2018). Phylogenomic analysis of 2556 single-copy protein-coding genes resolves most evolutionary relationships for the major clades in the most diverse group of lichen-forming fungi. Fungal Divers. 92, 31–41. doi: 10.1007/s13225-018-0407-7

[ref30] Ruprecht U. Fernández-Mendoza F. Türk R. Fryday A. M. (2020). High levels of endemism and local differentiation in the fungal and algal symbionts of saxicolous lecideoid lichens along a latitudinal gradient in southern South America. Lichenologist 52, 287–303. doi: 10.1017/S0024282920000225, PMID: 32788813 PMC7396322

[ref31] Smith K. Hill J. (2019). Defining the nature of blended learning through its depiction in current research. High. Educ. Res. Dev. 38, 383–397. doi: 10.1080/07294360.2018.1517732

[ref32] Tamura K. Stecher G. Kumar S. (2021). MEGA11 molecular evolutionary genetics analysis version 11. Mol. Biol. Evol. 38, 3022–3027. doi: 10.1093/molbev/msab120, PMID: 33892491 PMC8233496

[ref33] Tillich M. Lehwark P. Pellizzer T. Ulbricht-Jones E. S. Fischer A. Bock R.. (2017). GeSeq - versatile and accurate annotation of organelle genomes. Nucleic Acids Res. 45, W6–W11. doi: 10.1093/nar/gkx391, PMID: 28486635 PMC5570176

[ref9002] Valach M. Burger G. Gray M. W. Lang B. F. (2014). Widespread occurrence of organelle genome-encoded 5S rRNAs including permuted molecules. Nucleic Acids Res. 42, 13764–13777. doi: 10.1093/nar/gku1266 25429974 PMC4267664

[ref34] Wijayawardene N. N. Hyde K. D. Dai D. Q. Sánchez-García M. Goto B. T. Saxena R. K.. (2022). Outline of and fungus-like taxa-2021. Mycosphere 13, 53–453. doi: 10.5943/mycosphere/13/1/2

[ref35] Xavier B. B. Miao V. P. W. Jónsson Z. O. Andrésson O. S. (2012). Mitochondrial genomes from the lichenized fungi and: features and phylogeny. Fungal Biol. 116, 802–814. doi: 10.1016/j.funbio.2012.04.013 22749167

[ref36] Zhang D. Gao F. L. Jakovlic I. Zou H. Zhang J. Li W. X.. (2020). Phylosuite: an integrated and scalable desktop platform for streamlined molecular sequence data management and evolutionary phylogenetics studies. Mol. Ecol. Resour. 20, 348–355. doi: 10.1111/1755-0998.13096 31599058

[ref37] Zhang Y. J. Zhang S. Zhang G. Z. Liu X. Z. Wang C. S. Xu J. P. (2015). Comparison of mitochondrial genomes provides insights into intron dynamics and evolution in the caterpillar fungus. Fungal Genet. Biol. 77, 95–107. doi: 10.1016/j.fgb.2015.04.009 25896956

