# Supplementary material for: Comparative mitogenomics analysis of Peltigera species and new insights into the lichen phylogenetics
Source: Front Microbiol. 2025 Jul 15;16:1599036. doi: 10.3389/fmicb.2025.1599036 (PMC12305817; doi:10.3389/fmicb.2025.1599036)
Supplement: Supplementary file 1 [file Table_1.docx]

Supplementary Material

**Supplementary Table 1 Specimen collection information**

| Species name | Latitude | Attitude | Elvation | GenBank |
| --- | --- | --- | --- | --- |
| *Peltigera aphthosa* | 48°33'45" | 87°25'30" | 1340 m | PP740896 |
| *Peltigera continentalis* | 43°48'30" | 87°09'55" | 1950 m | PP740889 |
| *Peltigera degenii* | 43°45'71" | 87°09'28" | 1880 m | PP740898 |
| *Peltigera hymenina* | 48°33'45" | 87°25'30" | 1340 m | PP740897 |
| *Peltigera lepidophora* | 46°46′59′′ | 90°36′16″ | 1610 m | PP740891 |
| *Peltigera leucophlebia* | 48°33′45′′ | 87°25′30″ | 1045 m | PP740893 |
| *Peltigera malacea* | 48°23′51′′ | 87°12′6″ | 1830 m | PP740899 |
| *Peltigera polydactylon* | 43°13'0" | 84°18'52" | 1877 m | PP740890 |
| *Peltigera praetextata* | 45°47'18" | 82°54'39" | 2189 m | PP740892 |
| *Peltigera rufescens* | 43°48′33′′ | 87°38′57″ | 1946 m | PP740895 |
| *Peltigera venosa* | 45°47'15" | 82°54'33" | 2199 m | PP740894 |

**Supplementary Table 2 Specimen information downloaded from NCBI**

| Species name | GenBank |
| --- | --- |
| *Peltigera elisabethae* | OR468759 |
| *Peltigera neocanina* | OR473628 |
| *Peltigera canina* | OR470683 |
| *Peltigera ponojensis* | OR468758 |
| *Peltigera neckeri* | OR468736 |

**Supplementary Table 3 The size and proportion of protein-coding region, intergenic region, intronic region and RNA region in 11 mitochondrial genomes**

| Species | Protein-coding region (bp) | Intergenic region (bp) | Intronic region (bp) | RNA region (bp) |
| --- | --- | --- | --- | --- |
| *P. aphthosa* | 14,016 (21.26%) | 19,497 (29.58%) | 25,859 (39.23%) | 6,546 (9.93%) |
| *P. continentalis* | 14,169 (24.57%) | 20,921 (36.27%) | 15,660 (27.15%) | 6,926 (12.01%) |
| *P. degenii* | 14,130 (22.40%) | 25,690 (37.09%) | 22,746 (32.84%) | 6,701 (9.67%) |
| *P. hymenina* | 13,965 (26.80%) | 23,424 (44.95%) | 8,207 (15.75%) | 6,511 (12.50%) |
| *P. lepidophora* | 13,845 (19.10%) | 27,574 (38.04%) | 24,524 (33.83%) | 6,548 (9.03%) |
| *P. leucophlebia* | 14,121 (18.49%) | 28,856 (37.79%) | 26,929 (35.27%) | 6,447 (8.44%) |
| *P. malacea* | 13,821 (24.62%) | 18,738 (33.38%) | 16,641 (29.64%) | 6,938 (12.36%) |
| *P. polydactylon* | 13,971 (23.59%) | 25,318 (42.76%) | 13,086 (22.10%) | 6,839 (11.55%) |
| *P. preatextata* | 14,130 (21.09%) | 24,733 (36.91%) | 21,444 (32.00%) | 6,702 (10.00%) |
| *P. rufescens* | 14,034 (23.34%) | 21,991 (36.57%) | 17,254 (28.70%) | 6,849 (11.39%) |
| *p. venosa* | 13,782 (19.96%) | 23,541 (34.09%) | 25,957 (37.59%) | 5,782 (8.37%) |

**Supplementary Table 4 *P. aphthosa* mitochondrial genome Blastn with itself**

| Species | Similarity (%) | Aligned length /bp | Mismatched bases /bp | Gaps /bp | Start | End | Start | End | E value |
| --- | --- | --- | --- | --- | --- | --- | --- | --- | --- |
| *P. aphthosa* | 100 | 1501 | 0 | 0 | 39046 | 40546 | 37667 | 39167 | 0 |
| *P. aphthosa* | 100 | 1501 | 0 | 0 | 37667 | 39167 | 39046 | 40546 | 0 |
| *P. aphthosa* | 100 | 122 | 0 | 0 | 40425 | 40546 | 37667 | 37788 | 3.03E-59 |
| *P. aphthosa* | 100 | 122 | 0 | 0 | 37667 | 37788 | 40425 | 40546 | 3.03E-59 |
| *P. aphthosa* | 90.698 | 129 | 7 | 1 | 29356 | 29479 | 20842 | 20970 | 1.86E-41 |
| *P. aphthosa* | 90.698 | 129 | 7 | 1 | 20842 | 20970 | 29356 | 29479 | 1.86E-41 |
| *P. aphthosa* | 81.281 | 203 | 23 | 10 | 35324 | 35513 | 34638 | 34838 | 1.87E-36 |
| *P. aphthosa* | 81.281 | 203 | 23 | 10 | 34638 | 34838 | 35324 | 35513 | 1.87E-36 |
| *P. aphthosa* | 82.424 | 165 | 28 | 1 | 61171 | 61334 | 23334 | 23170 | 3.14E-34 |
| *P. aphthosa* | 82.424 | 165 | 28 | 1 | 23170 | 23334 | 61334 | 61171 | 3.14E-34 |
| *P. aphthosa* | 89.189 | 74 | 8 | 0 | 27838 | 27911 | 27773 | 27846 | 3.20E-19 |
| *P. aphthosa* | 89.189 | 74 | 8 | 0 | 27773 | 27846 | 27838 | 27911 | 3.20E-19 |

**Supplementary Table 5 *P. continentalis* mitochondrial genome Blastn with itself**

| Species | Similarity (%) | Aligned length /bp | Mismatched bases /bp | Gaps /bp | Start | End | Start | End | E value |
| --- | --- | --- | --- | --- | --- | --- | --- | --- | --- |
| *P. continentalis* | 84.521 | 407 | 59 | 3 | 52225 | 52629 | 18079 | 18483 | 1.34E-111 |
| *P. continentalis* | 84.521 | 407 | 59 | 3 | 18079 | 18483 | 52225 | 52629 | 1.34E-111 |
| *P. continentalis* | 98 | 200 | 4 | 0 | 27827 | 28026 | 7527 | 7726 | 4.91E-96 |
| *P. continentalis* | 98 | 200 | 4 | 0 | 7527 | 7726 | 27827 | 28026 | 4.91E-96 |
| *P. continentalis* | 81.298 | 262 | 45 | 4 | 52794 | 53053 | 18576 | 18835 | 2.42E-54 |
| *P. continentalis* | 81.298 | 262 | 45 | 4 | 18576 | 18835 | 52794 | 53053 | 2.42E-54 |
| *P. continentalis* | 95.413 | 109 | 1 | 2 | 34551 | 34656 | 33182 | 33289 | 1.14E-42 |
| *P. continentalis* | 95.413 | 109 | 1 | 2 | 33182 | 33289 | 34551 | 34656 | 1.14E-42 |
| *P. continentalis* | 96.471 | 85 | 3 | 0 | 27992 | 28076 | 14481 | 14565 | 8.94E-34 |
| *P. continentalis* | 96.471 | 85 | 3 | 0 | 14481 | 14565 | 27992 | 28076 | 8.94E-34 |
| *P. continentalis* | 90 | 80 | 8 | 0 | 58279 | 58358 | 16305 | 16384 | 1.17E-22 |
| *P. continentalis* | 90 | 80 | 8 | 0 | 16305 | 16384 | 58279 | 58358 | 1.17E-22 |

**Supplementary Table 6 *P. degenii* mitochondrial genome Blastn with itself**

| Species | Similarity (%) | Aligned length /bp | Mismatched bases /bp | Gaps /bp | Start | End | Start | End | E value |
| --- | --- | --- | --- | --- | --- | --- | --- | --- | --- |
| *P. degenii* | 91.133 | 203 | 16 | 2 | 30415 | 30616 | 9276 | 9477 | 1.18E-73 |
| *P. degenii* | 91.133 | 203 | 16 | 2 | 30415 | 30616 | 9276 | 9477 | 1.18E-73 |
| *P. degenii* | 91.133 | 203 | 16 | 2 | 9276 | 9477 | 30415 | 30616 | 1.18E-73 |
| *P. degenii* | 99.194 | 124 | 0 | 1 | 40194 | 40317 | 38821 | 38943 | 4.32E-58 |
| *P. degenii* | 99.194 | 124 | 0 | 1 | 38821 | 38943 | 40194 | 40317 | 4.32E-58 |
| *P. degenii* | 80.788 | 203 | 24 | 8 | 36487 | 36676 | 34649 | 34849 | 9.63E-35 |
| *P. degenii* | 80.788 | 203 | 24 | 8 | 34649 | 34849 | 36487 | 36676 | 9.63E-35 |
| *P. degenii* | 80.723 | 166 | 18 | 12 | 63766 | 63922 | 16742 | 16902 | 2.10E-26 |
| *P. degenii* | 80.723 | 166 | 18 | 12 | 16742 | 16902 | 63766 | 63922 | 2.10E-26 |
| *P. degenii* | 80 | 175 | 22 | 9 | 68510 | 68674 | 68457 | 68628 | 2.10E-26 |
| *P. degenii* | 80 | 175 | 22 | 9 | 68457 | 68628 | 68510 | 68674 | 2.10E-26 |
| *P. degenii* | 86.538 | 104 | 14 | 0 | 68774 | 68877 | 18063 | 18166 | 7.55E-26 |
| *P. degenii* | 86.538 | 104 | 14 | 0 | 18063 | 18166 | 68774 | 68877 | 7.55E-26 |

**Supplementary Table 7 *P. hymenina* mitochondrial genome Blastn with itself**

| Species | Similarity (%) | Aligned length /bp | Mismatched bases /bp | Gaps /bp | Start | End | Start | End | E value |
| --- | --- | --- | --- | --- | --- | --- | --- | --- | --- |
| *P. hymenina* | 97.902 | 143 | 2 | 1 | 15220 | 15361 | 6518 | 6660 | 1.45E-65 |
| *P. hymenina* | 97.902 | 143 | 2 | 1 | 6518 | 6660 | 15220 | 15361 | 1.45E-65 |
| *P. hymenina* | 97.692 | 130 | 3 | 0 | 21802 | 21931 | 20424 | 20553 | 6.80E-59 |
| *P. hymenina* | 97.692 | 130 | 3 | 0 | 20424 | 20553 | 21802 | 21931 | 6.80E-59 |
| *P. hymenina* | 96.522 | 115 | 3 | 1 | 15098 | 15212 | 3603 | 3716 | 2.48E-48 |
| *P. hymenina* | 96.522 | 115 | 3 | 1 | 3603 | 3716 | 15098 | 15212 | 2.48E-48 |
| *P. hymenina* | 88.732 | 71 | 3 | 4 | 43735 | 43804 | 42755 | 42821 | 4.33E-16 |
| *P. hymenina* | 88.732 | 71 | 3 | 4 | 42755 | 42821 | 43735 | 43804 | 4.33E-16 |

**Supplementary Table 8 *P. lepidophora* mitochondrial genome Blastn with itself**

| Species | Similarity (%) | Aligned length /bp | Mismatched bases /bp | Gaps /bp | Start | End | Start | End | E value |
| --- | --- | --- | --- | --- | --- | --- | --- | --- | --- |
| *P. lepidophora* | 100 | 56139 | 0 | 0 | 1 | 56139 | 1 | 56139 | 1.04E+05 |
| *P. lepidophora* | 94.059 | 202 | 10 | 2 | 24723 | 24924 | 7296 | 7495 | 2.74E-83 |
| *P. lepidophora* | 94.059 | 202 | 10 | 2 | 7296 | 7495 | 24723 | 24924 | 2.74E-83 |
| *P. lepidophora* | 91.579 | 190 | 9 | 6 | 30737 | 30925 | 29151 | 29334 | 2.80E-68 |
| *P. lepidophora* | 91.579 | 190 | 9 | 6 | 29151 | 29334 | 30737 | 30925 | 2.80E-68 |
| *P. lepidophora* | 96.032 | 126 | 3 | 2 | 33704 | 33829 | 32330 | 32453 | 1.03E-52 |
| *P. lepidophora* | 96.032 | 126 | 3 | 2 | 32330 | 32453 | 33704 | 33829 | 1.03E-52 |
| *P. lepidophora* | 92.222 | 90 | 7 | 0 | 24881 | 24970 | 14248 | 14337 | 6.37E-30 |
| *P. lepidophora* | 92.222 | 90 | 7 | 0 | 14248 | 14337 | 24881 | 24970 | 6.37E-30 |
| *P. lepidophora* | 91.304 | 69 | 4 | 2 | 55355 | 55422 | 55245 | 55312 | 2.32E-19 |

**Supplementary Table 9 *P. leucophlebia* mitochondrial genome Blastn with itself**

| Species | Similarity (%) | Aligned length /bp | Mismatched bases /bp | Gaps /bp | Start | End | Start | End | E value |
| --- | --- | --- | --- | --- | --- | --- | --- | --- | --- |
| *P. leucophlebia* | 88.926 | 298 | 30 | 1 | 71346 | 71643 | 28849 | 28555 | 8.25E-101 |
| *P. leucophlebia* | 88.926 | 298 | 30 | 1 | 28555 | 28849 | 71643 | 71346 | 8.25E-101 |
| *P. leucophlebia* | 100 | 124 | 0 | 0 | 44724 | 44847 | 43351 | 43474 | 3.14E-60 |
| *P. leucophlebia* | 100 | 124 | 0 | 0 | 43351 | 43474 | 44724 | 44847 | 3.14E-60 |
| *P. leucophlebia* | 84.694 | 196 | 25 | 3 | 75573 | 75766 | 75534 | 75726 | 1.48E-48 |
| *P. leucophlebia* | 84.694 | 196 | 25 | 3 | 75534 | 75726 | 75573 | 75766 | 1.48E-48 |
| *P. leucophlebia* | 90.4 | 125 | 9 | 3 | 36427 | 36549 | 12271 | 12394 | 1.16E-39 |
| *P. leucophlebia* | 90.4 | 125 | 9 | 3 | 12271 | 12394 | 36427 | 36549 | 1.16E-39 |
| *P. leucophlebia* | 100 | 79 | 0 | 0 | 75800 | 75878 | 23485 | 23563 | 3.25E-35 |
| *P. leucophlebia* | 100 | 79 | 0 | 0 | 23485 | 23563 | 75800 | 75878 | 3.25E-35 |
| *P. leucophlebia* | 84.247 | 146 | 19 | 3 | 75625 | 75769 | 75547 | 75689 | 5.44E-33 |
| *P. leucophlebia* | 84.247 | 146 | 19 | 3 | 75547 | 75689 | 75625 | 75769 | 5.44E-33 |
| *P. leucophlebia* | 90.385 | 104 | 9 | 1 | 36529 | 36632 | 21697 | 21799 | 7.04E-32 |

**Supplementary Table 9 *P. leucophlebia* mitochondrial genome Blastn with itself**

| Species | Similarity (%) | Aligned length /bp | Mismatched bases /bp | Gaps /bp | Start | End | Start | End | E value |
| --- | --- | --- | --- | --- | --- | --- | --- | --- | --- |
| *P. leucophlebia* | 90.385 | 104 | 9 | 1 | 21697 | 21799 | 36529 | 36632 | 7.04E-32 |
| *P. leucophlebia* | 84.783 | 92 | 11 | 3 | 74539 | 74628 | 63294 | 63384 | 5.56E-18 |
| *P. leucophlebia* | 84.783 | 92 | 11 | 3 | 63294 | 63384 | 74539 | 74628 | 5.56E-18 |
| *P. leucophlebia* | 100 | 36 | 0 | 0 | 54271 | 54306 | 54306 | 54271 | 2.61E-11 |

**Supplementary Table 10 *P. malacea* mitochondrial genome Blastn with itself**

| Species | Similarity (%) | Aligned length /bp | Mismatched bases /bp | Gaps /bp | Start | End | Start | End | E value |
| --- | --- | --- | --- | --- | --- | --- | --- | --- | --- |
| *P. malacea* | 100 | 72491 | 0 | 0 | 1 | 72491 | 1 | 72491 | 1.34E+05 |
| *P. malacea* | 96.142 | 337 | 11 | 2 | 61285 | 61620 | 24383 | 24718 | 1.91E-156 |
| *P. malacea* | 96.142 | 337 | 11 | 2 | 24383 | 24718 | 61285 | 61620 | 1.91E-156 |
| *P. malacea* | 100 | 122 | 0 | 0 | 40115 | 40236 | 38736 | 38857 | 3.66E-59 |
| *P. malacea* | 100 | 122 | 0 | 0 | 38736 | 38857 | 40115 | 40236 | 3.66E-59 |
| *P. malacea* | 91.473 | 129 | 6 | 1 | 30425 | 30548 | 21912 | 22040 | 4.84E-43 |

**Supplementary Table 10 *P. malacea* mitochondrial genome Blastn with itself**

| Species | Similarity (%) | Aligned length /bp | Mismatched bases /bp | Gaps /bp | Start | End | Start | End | E value |
| --- | --- | --- | --- | --- | --- | --- | --- | --- | --- |
| *P. malacea* | 91.473 | 129 | 6 | 1 | 21912 | 22040 | 30425 | 30548 | 4.84E-43 |
| *P. malacea* | 81.281 | 203 | 23 | 10 | 36393 | 36582 | 35707 | 35907 | 2.27E-36 |
| *P. malacea* | 81.281 | 203 | 23 | 10 | 35707 | 35907 | 36393 | 36582 | 2.27E-36 |
| *P. malacea* | 86.508 | 126 | 10 | 6 | 15305 | 15423 | 15236 | 15361 | 8.21E-31 |
| *P. malacea* | 86.508 | 126 | 10 | 6 | 15236 | 15361 | 15305 | 15423 | 8.21E-31 |
| *P. malacea* | 91.892 | 74 | 5 | 1 | 68940 | 69012 | 62841 | 62768 | 6.44E-22 |
| *P. malacea* | 91.892 | 74 | 5 | 1 | 62768 | 62841 | 69012 | 68940 | 6.44E-22 |
| *P. malacea* | 88 | 75 | 9 | 0 | 28907 | 28981 | 28842 | 28916 | 5.01E-18 |
| *P. malacea* | 88 | 75 | 9 | 0 | 28842 | 28916 | 28907 | 28981 | 5.01E-18 |
| *P. malacea* | 100 | 44 | 0 | 0 | 62381 | 62424 | 62424 | 62381 | 8.39E-16 |

**Supplementary Table 11 *P. polydactylon* mitochondrial genome Blastn with itself**

| Species | Similarity (%) | Aligned length /bp | Mismatched bases /bp | Gaps /bp | Start | End | Start | End | E value |
| --- | --- | --- | --- | --- | --- | --- | --- | --- | --- |
| *P. polydactylon* | 100 | 110 | 0 | 0 | 22957 | 23066 | 11213 | 11322 | 1.14E-52 |
| *P. polydactylon* | 100 | 110 | 0 | 0 | 11213 | 11322 | 22957 | 23066 | 1.14E-52 |
| *P. polydactylon* | 96.396 | 111 | 4 | 0 | 29525 | 29635 | 28148 | 28258 | 1.49E-46 |
| *P. polydactylon* | 96.396 | 111 | 4 | 0 | 28148 | 28258 | 29525 | 29635 | 1.49E-46 |
| *P. polydactylon* | 97.531 | 81 | 2 | 0 | 58728 | 58808 | 13104 | 13184 | 3.27E-33 |
| *P. polydactylon* | 97.531 | 81 | 2 | 0 | 13104 | 13184 | 58728 | 58808 | 3.27E-33 |
| *P. polydactylon* | 85.333 | 75 | 11 | 0 | 21453 | 21527 | 21388 | 21462 | 7.24E-15 |
| *P. polydactylon* | 85.333 | 75 | 11 | 0 | 21388 | 21462 | 21453 | 21527 | 7.24E-15 |

**Supplementary Table 12 *P. praetextata* mitochondrial genome Blastn with itself**

| Species | Similarity (%) | Aligned length /bp | Mismatched bases /bp | Gaps /bp | Start | End | Start | End | E value |
| --- | --- | --- | --- | --- | --- | --- | --- | --- | --- |
| *P. praetextata* | 91.133 | 203 | 16 | 2 | 29148 | 29349 | 9280 | 9481 | 1.10E-73 |
| *P. praetextata* | 91.133 | 203 | 16 | 2 | 9280 | 9481 | 29148 | 29349 | 1.10E-73 |
| *P. praetextata* | 98.387 | 124 | 0 | 1 | 38939 | 39060 | 37565 | 37688 | 1.88E-56 |
| *P. praetextata* | 98.387 | 124 | 0 | 1 | 37565 | 37688 | 38939 | 39060 | 1.88E-56 |
| *P. praetextata* | 80.788 | 203 | 24 | 8 | 35231 | 35420 | 33382 | 33582 | 9.01E-35 |
| *P. praetextata* | 80.788 | 203 | 24 | 8 | 33382 | 33582 | 35231 | 35420 | 9.01E-35 |
| *P. praetextata* | 80.723 | 166 | 18 | 12 | 61453 | 61609 | 16740 | 16900 | 1.96E-26 |
| *P. praetextata* | 80.723 | 166 | 18 | 12 | 16740 | 16900 | 61453 | 61609 | 1.96E-26 |
| *P. praetextata* | 86.538 | 104 | 14 | 0 | 66510 | 66613 | 18058 | 18161 | 7.07E-26 |
| *P. praetextata* | 86.538 | 104 | 14 | 0 | 18058 | 18161 | 66510 | 66613 | 7.07E-26 |
| *P. praetextata* | 76.19 | 210 | 31 | 13 | 66212 | 66410 | 66163 | 66364 | 3.31E-19 |
| *P. praetextata* | 76.19 | 210 | 31 | 13 | 66163 | 66364 | 66212 | 66410 | 3.31E-19 |

**Supplementary Table 13 *P. rufescens* mitochondrial genome Blastn with itself**

| Species | Similarity (%) | Aligned length /bp | Mismatched bases /bp | Gaps /bp | Start | End | Start | End | E value |
| --- | --- | --- | --- | --- | --- | --- | --- | --- | --- |
| *P. rufescens* | 99.167 | 120 | 1 | 0 | 34071 | 34190 | 32703 | 32822 | 1.52E-56 |
| *P. rufescens* | 99.167 | 120 | 1 | 0 | 32703 | 32822 | 34071 | 34190 | 1.52E-56 |
| *P. rufescens* | 96.296 | 81 | 3 | 0 | 25920 | 26000 | 15564 | 15644 | 1.57E-31 |
| *P. rufescens* | 96.296 | 81 | 3 | 0 | 15564 | 15644 | 25920 | 26000 | 1.57E-31 |
| *P. rufescens* | 80.226 | 177 | 20 | 13 | 57444 | 57610 | 16018 | 16189 | 4.40E-27 |
| *P. rufescens* | 80.226 | 177 | 20 | 13 | 16018 | 16189 | 57444 | 57610 | 4.40E-27 |
| *P. rufescens* | 96.97 | 66 | 2 | 0 | 25889 | 25954 | 8707 | 8772 | 7.36E-25 |
| *P. rufescens* | 96.97 | 66 | 2 | 0 | 8707 | 8772 | 25889 | 25954 | 7.36E-25 |
| *P. rufescens* | 86.139 | 101 | 10 | 3 | 59647 | 59746 | 17357 | 17454 | 3.42E-23 |
| *P. rufescens* | 86.139 | 101 | 10 | 3 | 17357 | 17454 | 59647 | 59746 | 3.42E-23 |

**Supplementary Table 14 *P. venosa* mitochondrial genome Blastn with itself**

| Species | Similarity (%) | Aligned length /bp | Mismatched bases /bp | Gaps /bp | Start | End | Start | End | E value |
| --- | --- | --- | --- | --- | --- | --- | --- | --- | --- |
| *P. venosa* | 97.561 | 123 | 3 | 0 | 38253 | 38375 | 36884 | 37006 | 9.30E-55 |
| *P. venosa* | 97.561 | 123 | 3 | 0 | 36884 | 37006 | 38253 | 38375 | 9.30E-55 |
| *P. venosa* | 92.405 | 79 | 3 | 2 | 68510 | 68588 | 23275 | 23350 | 3.49E-24 |
| *P. venosa* | 92.405 | 79 | 3 | 2 | 23275 | 23350 | 68510 | 68588 | 3.49E-24 |
| *P. venosa* | 91.837 | 49 | 2 | 2 | 31765 | 31811 | 12682 | 12730 | 2.13E-11 |
| *P. venosa* | 91.837 | 49 | 2 | 2 | 12682 | 12730 | 31765 | 31811 | 2.13E-11 |

**Supplementary Table 15 Initiation and termination codons of 11 mitochondrial protein-coding genes in the *Peltigera***

| Species  Gene | *atp6* | *atp8* | *atp9* | *cytb* | *cox1* | *cox2* | *cox3* | *nad1* | *nad2* | *nad3* | *nad4* | *nad4L* | *nad5* | *nad6* | *rps3* |
| --- | --- | --- | --- | --- | --- | --- | --- | --- | --- | --- | --- | --- | --- | --- | --- |
|  | Start  End | Start  End | Start  End | Start  End | Start  End | Start  End | Start  End | Start  End | Start  End | Start  End | Start  End | Start  End | Start  End | Start  End | Start  End |
| *P. aphthosa* | ATG  TAA | ATG  TAA | ATG  TAA | GTG  GAC | TTA  TAA | ATT  TAG | ATT  TAG | ATG  TAA | ATG  TAA | ATG  TAA | ATG  TAA | ATG  TAA | ATG  TAA | ATA  TAA | ATG  TAA |
| *P. continentalis* | ATG  TAA | ATG  TAA | ATG  TAA | GTG  GAC | ATG  TAA | ATT  TAG | ATT  TAG | ATG  TAA | ATG  TAA | ATG  TAA | ATG  TAA | ATG  TAA | ATG  TAA | ATA  TAA | ATG  TAA |
| *P. degenii* | ATG  TAA | ATG  TAA | ATG  TAA | GTG  GAC | ATG  TAA | ATT  TAG | ATT  TAG | ATG  TAA | ATG  TAA | ATG  TAA | ATG  TAA | ATG  TAA | ATG  TAA | ATA  TAA | ATG  TAA |
| *P. hymenina* | ATG  TAA | ATG  TAA | ATG  TAA | GTG  GAC | ATG  TAG | ATT  TAG | ATT  TAG | ATG  TAA | ATG  TAA | ATG  TAA | ATG  TAA | ATG  TAA | ATG  TAA | ATG  TAA | ATG  TAA |
| *P. lepidophora* | ATG  TAA | ATG  TAA | ATG  TAA | GTG  GAC | TTA  TAA | ATT  TAG | ATT  TAG | ATG  TAA | ATG  TAA | ATG  TAA | ATG  TAA | ATG  TAA | ATG  TAA | ATA  TAA | ATG  TAA |
| *P. leucophlebia* | ATG  TAA | ATG  TAA | ATG  TAA | GTG  GAC | TTA  TAA | ATT  TAG | ATT  TAG | ATG  TAA | ATG  TAA | ATG  TAA | ATG  TAA | ATG  TAA | ATG  TAA | ATA  TAA | ATG  TAA |

| Species  Gene | *atp6* | *atp8* | *atp9* | *cytb* | *cox1* | *cox2* | *cox3* | *nad1* | *nad2* | *nad3* | *nad4* | *nad4L* | *nad5* | *nad6* | *rps3* |
| --- | --- | --- | --- | --- | --- | --- | --- | --- | --- | --- | --- | --- | --- | --- | --- |
|  | Start  End | Start  End | Start  End | Start  End | Start  End | Start  End | Start  End | Start  End | Start  End | Start  End | Start  End | Start  End | Start  End | Start  End | Start  End |
| *P. malacea* | ATG  TAA | ATG  TAA | ATG  TAA | GTG  GAC | TTA  TAG | ATT  TAG | ATT  TAG | ATG  TAA | ATG  TAA | ATG  TAA | ATG  TAA | ATG  TAA | ATG  TAA | ATA  TAA | ATG  TAA |
| *P. polydactylon* | ATG  TAA | ATG  TAA | ATG  TAA | GTG  GAC | ATG  TAG | ATT  TAG | ATT  TAG | ATG  TAA | ATG  TAA | ATG  TAA | ATG  TAA | ATG  TAA | ATG  TAA | ATG  TAA | ATG  TAA |
| *P. praetextata* | ATG  TAA | ATG  TAA | ATG  TAA | GTG  GAC | ATA  TAA | ATT  TAG | ATT  TAG | ATG  TAA | ATG  TAA | ATG  TAA | ATG  TAA | ATG  TAA | ATG  TAA | ATA  TAA | ATG  TAA |
| *P. rufescens* | ATG  TAA | ATG  TAA | ATG  TAA | GTG  GAC | TTA  TAA | ATT  TAG | ATT  TAG | ATG  TAA | ATG  TAA | ATG  TAA | ATG  TAA | ATG  TAA | ATG  TAA | ATA  TAA | ATG  TAA |
| *P. venosa* | ATG  TAA | ATG  TAA | ATG  TAA | GTG  GAC | ATG  TAA | ATT  TAG | ATT  TAG | ATG  TAA | ATG  TAA | ATG  TAA | ATG  TAA | ATG  TAA | ATG  TAA | ATA  TAA | ATG  TAA |

**Supplementary Table 15 Initiation and termination codons of 25 mitochondrial protein-coding genes in the *Peltigera***
